# A ROS-responsive synergistic delivery system for combined immunotherapy and chemotherapy

**DOI:** 10.1016/j.mtbio.2022.100284

**Published:** 2022-05-11

**Authors:** Doudou Hu, Wei Zhang, Jiajia Xiang, Dongdong Li, Yong Chen, Pengcheng Yuan, Shiqun Shao, Zhuxian Zhou, Youqing Shen, Jianbin Tang

**Affiliations:** a Zhejiang Key Laboratory of Smart BioMaterials and Center for Bionanoengineering, College of Chemical and Biological Engineering, Zhejiang University, Hangzhou, 310027, China; b ZJU-Hangzhou Global Scientific and Technological Innovation Center, Hangzhou, 311215, China; c Key Laboratory of Biomass Chemical Engineering of the Ministry of Education, College of Chemical and Biological Engineering, Zhejiang University, Hangzhou, 310027, China; d Subtropical Sericulture and Mulberry Resources Protection and Safety Engineering Research Center, College of Animal Science, South China Agricultural University, Guangzhou, Guangdong, 510642, China

**Keywords:** Immune checkpoint blockade, Immunotherapy, ROS-Responsive, Combination therapy, Synergistic effect

## Abstract

Immune checkpoint blockade (ICB) therapies that target programmed cell death-1 (PD-1)/programmed cell death-ligand 1 (PD-L1) pathway are currently used for the treatment of various cancer types. However, low response rates of ICB remain the major issue and limit their applications in clinic. Here, we developed a ROS-responsive synergistic delivery system (pep-PAPM@PTX) by integrating physically-encapsulated paclitaxel (PTX) and surface-modified anti-PD-L1 peptide (pep) for combined chemotherapy and ICB therapy. Pep-PAPM@PTX could bind the cell surface PD-L1 and drive its recycling to lysosomal degradation, thus reverting PTX-induced PD-L1 upregulation and downregulating PD-L1 expression. As a result, pep-PAPM@PTX significantly promoted T cell infiltration and increased tumor immunoactivating factors, synergizing PTX chemotherapy to achieve enhanced anticancer potency in a triple-negative breast cancer (TNBC) model.

## Graphical abstract

A ROS-responsive synergistic delivery system (pep-PAPM@PTX) was developed by integrating physically-encapsulated paclitaxel (PTX) and surface-modified anti-PD-L1 peptide (pep) for combined chemotherapy and ICB therapy.

**Figure:**
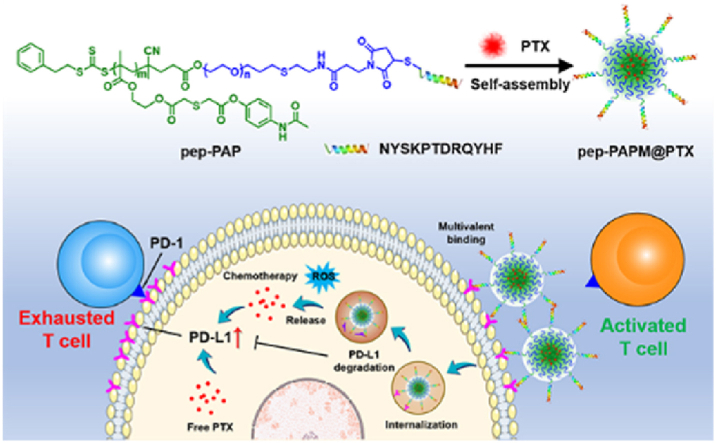

## 1 Introduction

Immunotherapy, especially immune checkpoint blockade (ICB) with antibodies targeting the PD-1/PD-L1 pathway, has revolutionized cancer treatment in the past decade [1]. However, monoclonal antibody drugs are perplexed by the high production cost, low response rate, and inherent immunogenicity; only a subset of patients can benefit from this therapy [2]. In contrast to antibodies of ICB, synthetic peptides offer advantages of easier production, higher stability, lower immunogenicity, and versatility in chemical modification and may have greater potential for stable clinical therapeutic windows and frequent administration [3]. Anti-PD-L1 peptides have been demonstrated as a promising alternative to antibodies for PD-1/PD-L1 axis blockade [[4], [5], [6]].

Despite the emergency of new treatments, chemotherapy is still most widely used in clinic, and promise to combine with immunotherapy to activate the immune microenvironment and promote immune responses [7,8]. Increasing evidence has shown that chemotherapeutic drugs, such as doxorubicin (DOX), oxaliplatin (OXA), and paclitaxel (PTX), can boost the antitumor immune response by eliciting considerable immunogenic cell death (ICD) of the tumor cells, thus facilitating the intratumoral infiltration of the cytotoxic T lymphocytes (CTLs) [9,10]. However, upon chemotherapy, the remaining tumor cells would upregulate PD-L1 expression to evade the immunosurveillance of T cells, weakening their functions and finally leading to exhaustion of the recruited T cells [11]. Although the prevalent PD-L1 blockade therapies with α-PD-L1, to some extent, conformationally block the PD-1/PD-L1 interaction axis, tumors cells can adaptively recycle PD-L1 after internalization of antibody-bound PD-L1 and repopulate them onto the cell surface [12,13]. Therefore, it is key for PD-L1 blockade therapy to prevent PD-L1 recycling and direct PD-L1 trafficking to lysosomal degradation.

Moreover, toxicity on the immune system and normal tissues induced by chemotherapy may cause systemic and intratumoral lymphocyte depletion, leading to an immunosuppressive state [14,15]. Since combination therapy may suffer a higher risk of adverse effects, the safety concerns of combined ICB and chemotherapy remain crucial in clinic [16,17]. Furthermore, immunotherapeutic and chemotherapeutic agents may have very different physicochemical properties and distinct pharmacokinetic profiles, making it challenging to efficiently transport them to the targets for combination therapy. Thus, it is urgently required to develop tumor-targeting delivery systems that integrate chemotherapeutic drugs with ICB agents to activate tumor-specific immune responses, thus achieving synergistic outcomes and reducing side effects [18,19].

**Scheme 1:**
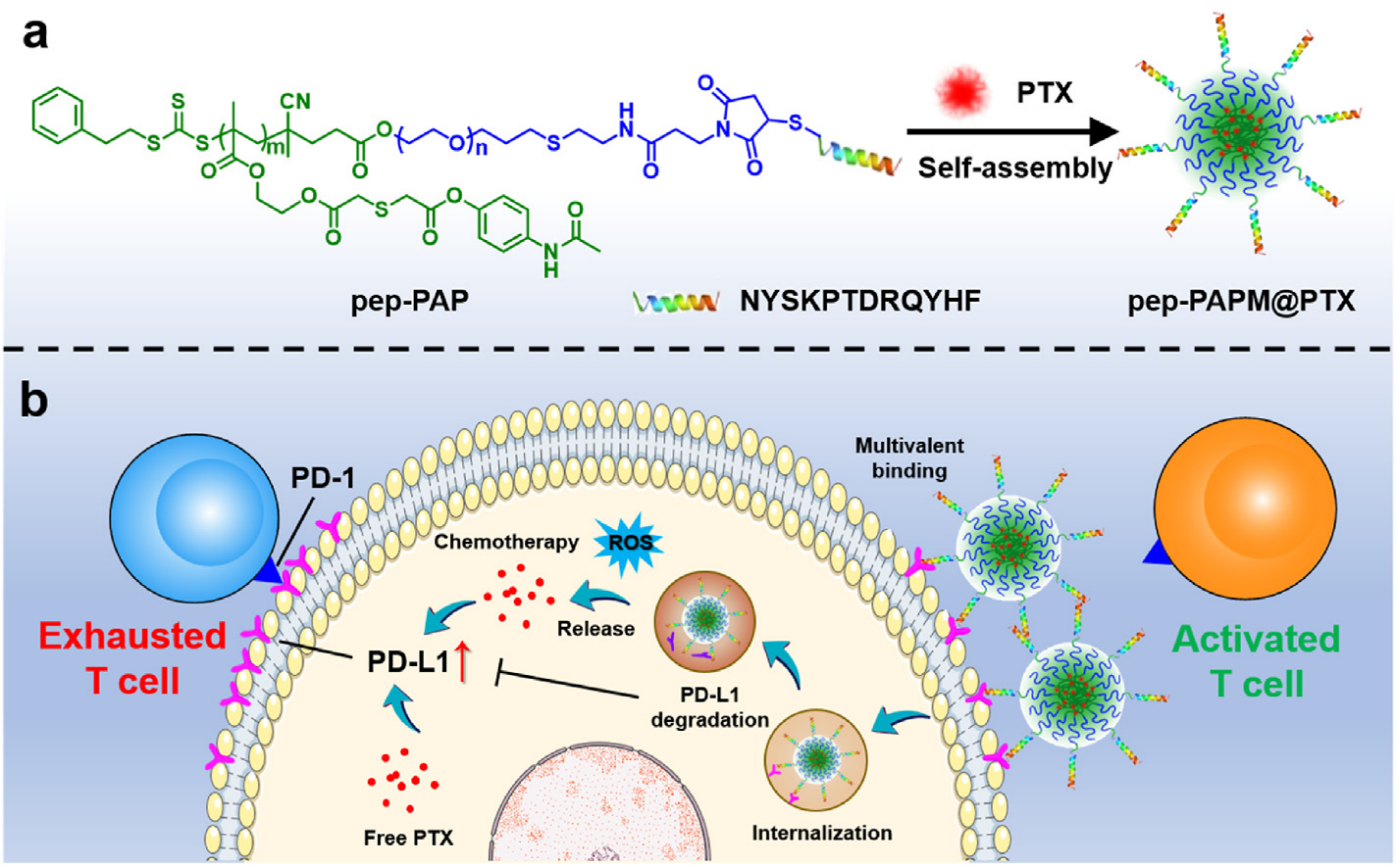
Schematic illustration of the PD-L1-targeting ROS-responsive micelle for combined immunotherapy and chemotherapy. (a) The anti-PD-L1 peptide modified amphiphilic block polymer pep-PAP self-assembled with PTX in water to form micelles (pep-PAPM@PTX). (b) pep-PAPM@PTX binded the cell surface PD-L1 multivalently and drove its recycling to lysosome degradation, thus downregulating PD-L1 expression. Meanwhile, pep-PAPM@PTX released PTX in response to elevated ROS levels, exerting cell-killing abilities to synergize immunotherapy.

In this study, we designed a ROS-responsive anti-PD-L1 peptide-functionalized block copolymer, pep-PAP, to co-assemble with paclitaxel (PTX), forming a synergistic drug delivery system (pep-PAPM@PTX) to combine cancer immunotherapy and chemotherapy (Scheme 1). The PD-L1-targeting D-peptide (NYSKPTDRQYHF, pep) [[20], [21], [22], [23]] on the micelle surface could bind the tumor cell surface PD-L1 via multivalent crosslinking, directing PD-L1 into lysosome degradation and thus downregulating the PD-L1 expression. Moreover, upon oxidization by the elevated ROS levels in tumor cells, pep-PAPM@PTX underwent micellar structure dissociation for fast PTX release. As a result, pep-PAPM@PTX dramatically promoted infiltration of cytotoxic T cells and secretion of tumor immunoactivating factors, thus synergizing PTX chemotherapy to augment anticancer efficacy in a TNBC model.

## 2 Materials and method

### 2.1 Materials

Paclitaxel (PTX) and allyloxypoly (ethylene glycol) with a molecular weight of ∼2000 (APEG_2k_-OH) were purchased from Sigma Aldrich (Shanghai, China). 4-Acetamidophenol, thiodiglycolic anhydride, 4-(dimethylamino)pyridine (DMAP), 2-hydroxyethyl methacrylate (HEMA), 1-ethyl-3-(3-dimethylaminopropyl)carbodiimide hydrochloride (EDC·HCl), cysteamine, 2,2-dimethoxy-2-phenylacetophenone (DMPA), N,N′-dicyclohexylcarbodiimide (DCC), azobisisobutyronitrile (AIBN), trifluoroacetic acid (TFA), and 3-(maleimido)propionic acid N-hydroxysuccinimide ester (MAL-NHS) were purchased from Energy Chemical (Shanghai, China). Anti-PD-L1 peptide CNYSKPTDRQYHF (pep) was purchased from Bankpeptide Biological Technology Co, Ltd (Hefei, China). All other organic reagents were purchased from Sinopharm Chemical Reagent Co, Ltd (Shanghai, China). APC-αPD-L1was purchased from Biolegend (USA). The chain transfer agent, PETTC, was synthesized as previously reported [24]. BALB/c mice were purchased from the SLAC Laboratory Animal Co, Ltd. (Shanghai, China). The animal experiments were approved by the Animal Care and Use Committee of Zhejiang University and were performed according to the guidelines.

### 2.2 Synthesis of block copolymer PAP and pep-PAP

#### 2.2.1 AP monomer

Acetaminophen (5.0 ​g, 30 ​mmol) and DMAP (0.8 ​g, 7 ​mmol) were mixed in tetrahydrofuran (THF), and thiodiglycolic anhydride (5.1 ​g, 38 ​mmol) was added into the solution under stirring. After stirring overnight at room temperature, HEMA (8.5 ​g, 43 ​mmol) and DMAP (1.6 ​g, 14 ​mmol) were added. EDC·HCl (9.5 ​g, 47 ​mmol) dissolved in 20 ​mL dichloromethane (DCM) was added dropwise to the mixture, and the reaction solution was stirred for another 24 ​h at room temperature. The solution was concentrated under reduced pressure, and then the residue was redissolved in DCM and washed with 1 ​N HCl (50 ​mL ​× ​3) and saturated brine. The crude product was further purified through a silica column (hexane: ethyl acetate ​= ​1:1) to give the product AP monomer as a white solid. ^1^H NMR (400 ​MHz, DCM): δ ​= ​10.02 (s, 1H), 7.60 (m, 2H), 7.06 (m, 2H), 6.04 (d, 1H), 5.69 (m, 1), 4.32 (d, 4H), 3.65 (d, 4H), 2.04 (s, 3H), 1.87 (s, 3H).

#### 2.2.2 BocNH-PEG-OH

APEG-OH (2.67 ​g, 1.3 ​mmol), 2-(BOC-amino)ethanethiol (1.18 ​g, 6 ​mmol), and DMPA (0.041 ​g, 0.16 ​mmol) were dissolved in methanol. The solution was bubbled with nitrogen for 30 ​min and sealed under a nitrogen atmosphere. Then the solution was exposed to UV light (5000 ​μW/cm^2^) for 12 ​h. After precipitating in ethyl ether and drying under a vacuum, the product BocNH-PEG-OH was obtained as a white solid. ^1^H NMR (400 ​MHz, DMSO): δ ​= ​6.92 (d, 1H), 4.58 (t, 1.5H), 3.68 (m, 1.5 ​H), 3.51 (s, 180 ​H), 1.37 (s, 8 ​H).

#### 2.2.3 BocNH-PEG-PETTC

PETTC (0.965 ​g, 2.5 ​mmol), BocNH-PEG-OH (1.9 ​g, 0.87 ​mmol), and DMAP (0.0684 ​g, 0.6 ​mmol) were dissolved in anhydrous DCM at 0 ​°C. DCC (0.855 ​g, 4.1 ​mmol) dissolved in 5 ​mL DCM was added dropwise. The solution was warmed to room temperature and stirred for 12 ​h. After filtration, the solution was concentrated under reduced pressure and precipitated in diethyl ether to obtain the macromolecular RAFT chain transfer agent BocNH-PEG-PETTC. ^1^H NMR (400 ​MHz, DMSO): δ ​= ​7.28 (m, 5H), 6.92 (s, 1H), 4.15 (m, 1H), 3.51 (s, 180H), 2.96 (m, 2H), 1.85 (s, 2.7H), 1.72 (m, 2H), 1.37 (s, 8H).

#### 2.2.4 Block copolymer PAP

Block copolymer PAP was synthesized via RAFT polymerization. Briefly, AP monomer (0.5 ​g, 1.27 ​mmol), BocNH-PEG-PETTC (0.304 ​g, 0.127 ​mmol), and AIBN (0.004 ​g, 0.025 ​mmol) were dissolved in 4 ​mL dimethylformamide (DMF) and charged into a Schlenk tube. The solution was degassed by purging with nitrogen for 15 ​min and then placed in an oil bath at 65 ​°C. After 13 ​h, the polymerization reaction was quenched with liquid nitrogen and exposed to air. After reprecipitation in ether, the block copolymer PAP was obtained as a light-yellow solid. ^1^H NMR (400 ​MHz, DMSO): δ ​= ​9.98 (s, 9H), 7.59 (d, 16H), 7.23 (d, 4H), 7.04 (d, 16H), 4.17 (d, 36H), 3.51 (s, 180H), 2 (d, 27H), 1.37 (s, 9H). The fluorescence-labeled PAP was synthesized as described above except for replacing 1% mol of AP monomer with Bodipy monomer.

#### 2.2.5 Pep-modified block copolymer pep-PAP

PAP was dissolved in anhydrous DCM and treated with TFA (3:1) for 2 ​h. The solvent was removed under reduced pressure. The deprotected PAP (0.6 ​g, 0.11 ​mmol), MAL-NHS (0.0855 ​g, 0.33 ​mmol), and triethylamine (0.2 ​mL) were dissolved in anhydrous DMF. After stirring at room temperature for 24 ​h, the solution was dialyzed against DMSO for 24 ​h to remove the excess MAL-NHS. pep (0.23 ​g, 0.138 ​mmol) was then added to the solution with a catalytic amount of tributylphosphane, and the mixture was stirred at room temperature for 24 ​h. The solution was dialyzed successively against DMSO and DI water and then lyophilized to give pep-PAP as a pale-yellow solid.

### 2.3 Fabrication of pep-PAPM and pep-PAPM@PTX

pep-PAP micelles (pep-PAPM) were co-assembled from PAP and pep-PAP by the nanoprecipitation method. Briefly, PAP and pep-PAP with different ratios were dissolved in DMSO. The solution was added dropwise to DI water with vigorous stirring for 5 ​min. The pep-PAPM was obtained by dialyzing the solution against DI water for 24 ​h. To prepare PTX-loaded PAPM and pep-PAPM, PTX and PAP/pep-PAP (1:10, w/w) were dissolved in DMSO and added dropwise to DI water with vigorous stirring. After dialyzing the solution against DI water, PAPM@PTX or pep-PAPM@PTX was obtained by filtering the solution with a 0.45 ​μm filter.

### 2.4 Characterization of pep-PAPM

The size was measured by dynamic light scattering (DLS) (ZetaSizer Nano-ZS90, Malvern Instruments Ltd, UK), and the morphology of the micelles was observed by transmission electron microscope (TEM) (JEM-1200EX, Japan).

The critical micelle concentration (CMC) of pep-PAPM was determined by Nile red fluorescence assay. Nile red (1 ​μM) in DCM was added to each vial, and the solvent was allowed to evaporate. 5 ​mL of micelle solution with concentrations varying from 0.001 to 1 ​mg/mL was transferred to each vial. The solution was vigorously stirred for 12 ​h in the dark at 37 ​°C. Then, the fluorescence intensity at 620 ​nm (579 ​nm excitation) was measured by a SpectraMax M2e reader and plotted as a function of micelle concentration.

### 2.5 Determination of PTX concentration and in vitro drug release by HPLC

The drug loading content (DLC) and encapsulation efficiency (EE) of pep-PAPM@PTX were assayed by HPLC using a C18 column (4.6 ​mm ​× ​250 ​mm, Waters, Ireland, 35 ​°C) with an isocratic elution of acetonitrile/DI water (60:40, v/v) at a flow rate of 1.0 ​mL/min. In vitro PTX release profiles were examined via the dialysis method. Briefly, 0.6 ​mL pep-PAPM solution (eq. dose of 1 ​mg/mL PTX) was added in a dialysis bag (MWCO ​= ​3500 ​Da) and incubated in 50 ​mL PBS with 0, 0.1 ​M, or 1 ​mM ​H_2_O_2_ containing 0.5% tween 80 to maintain sink conditions in a shaker (37 ​°C, 100 ​rpm/min). Samples (1 ​mL) were collected at predetermined time points, and equal volumes of fresh PBS were added. The PTX contents were measured by HPLC, and cumulative PTX release profiles were calculated.

### 2.6 In vitro uptake of pep-PAPM

For quantitative analysis of the cellular uptake, 4T1 cells were seeded at the density of 1 ​× ​10^6^ ​cells per well in 24-well plates and incubated for 24 ​h. The cells were incubated with FITC-labeled pep-PAPM for a specified time. After incubation, the cells were rinsed with PBS (pH 7.4) twice and collected by trypsin treatment. The harvested cells were suspended in PBS and centrifuged at 1000 ​rpm for 5 ​min. The supernatants were discarded, and the cell pellets were resuspended with PBS to obtain the cell suspension, which was analyzed by flow cytometry (BD FACSCalibur, USA). For confocal laser scanning microscopy (CLSM) observation of cellular uptake of pep-PAPM, 4T1 cells were seeded at the density of 1 ​× ​10^6^ ​cells on a glass-bottom dish. After incubation for 4 ​h with pep-PAPM, the cells were incubated with Hoechst33342 and LysoTracker Red for another 15 ​min before CLSM observation. The excitation wavelengths of Hoechst33342, FITC, and LysoTracker Red are 405 ​nm, 488 ​nm, and 543 ​nm, respectively.

### 2.7 Cytotoxicity assay

3-(4,5-Dimethythiazol-2-yl)-2,5-diphenyltetrazolium bromide (MTT) assays were used to assess the cytotoxicity of free PTX, pep-PAPM, and pep-PAPM@PTX in 4T1 cells. Briefly, cells were seeded in 96 well plates at a density of 5 ​× ​10^3^ ​cells per well and incubated overnight. Cells were exposed to serial dilutions of the drugs and cultivated for another 24 and 48 ​h, and then the medium was replaced by a fresh medium containing MTT. After a 3 h-incubation, the yellow tetrazolium salt (MTT) was metabolized into dark blue formazan crystals, and the medium was carefully removed. Finally, 0.1 ​mL of DMSO was added to each well, and the plate was gently shaken to dissolve the precipitates. The absorbance in each well was determined at 562 ​nm using a microplate spectrophotometer (Molecular Devices, SpectraMax M2e, USA). Cell viability was calculated as the ratio of the absorbance of the wells incubated with the drug to that of the wells incubated with a culture medium.

### 2.8 Intracellular ROS and PD-L1 expression

To determine the interactions between pep-PAPM and PD-L1 on the surface of 4T1 cells, 4T1 cells were incubated with different formulations (pep, PAPM, pep-PAPM) at 4 ​°C for 1 ​h. Then cells were washed with PBS and collected as cell suspensions. APC-αPD-L1 was added to the suspensions and incubated at room temperature in the dark. After 20 ​min, the cells were analyzed by flow cytometry. To observe the effect of drug-loaded micelles on PD-L1 expression, 4T1 cells were treated with free PTX, PAPM@PTX, and pep-PAPM@PTX (PTX concentration: 20 ​μg/mL) for 24 ​h at 37 ​°C. Then, the cells were treated as the method described above and analyzed by flow cytometry.

### 2.9 In vivo biodistribution

Female BALB/c mice bearing 4T1 breast tumor models were randomly divided into two groups (n ​= ​3). When the tumors reached about 100–200 ​mm^3^, Bodibpy labeled-PAPM and pep-PAPM were intravenously injected via the tail vein. Images were taken at 0–24 ​h after injection using the IVIS Spectrum Pre-clinical In Vivo Imaging System (Caliper Life Sciences, USA) with a 704 ​nm excitation wavelength and a 735 ​nm filter to collect the fluorescence signals of Bodipy. The mice were sacrificed after injection at 24 ​h, and tumors and main organs, including heart, liver, spleen, lung, and kidneys, were collected for imaging and biodistribution analysis. Results were analyzed using Living Image 4.3.1 software (Caliper Life Sciences).

In the drug biodistribution experiments, female BALB/c mice were inoculated with 4T1 tumors by subcutaneously injecting 1 ​× ​10^6^ ​cells. When the tumor volume reached 200 ​mm^3^, the mice were randomly divided into three groups (n ​= ​3) and treated with Taxol, PAPM@PTX, or pep-PAPM@PTX at a PTX-equivalent dose of 10 ​mg/kg and then sacrificed after 24 ​h post-treatment. Tumors and major organs (heart, liver, spleen, lung, kidneys) were excised and washed with PBS before being weighed. The organs or tissues were cut into small pieces and homogenized, and PTX was extracted with methanol. The supernatant was collected after centrifugation (5000 ​rpm, 10 ​min) and volatilized by nitrogen gas. The remaining was added 200 ​μL acetonitrile and centrifuged at 12,000 ​rpm for 10 ​min. The supernatant was subjected to HPLC to determine PTX levels, and the corresponding PTX tissue concentrations were calculated accordingly.

### 2.10 In vivo antitumor effect

Female BALB/c mice were inoculated subcutaneously with 4T1 cells (5 ​× ​10^5^). When the tumor volume reached about 50 ​mm^3^, the mice were randomly divided into three groups (n ​= ​5): (1) PBS control; (2) Taxol (PTX 10 ​mg/kg); (3) pep (6 ​mg/kg); (4) pep-PAPM (pep 6 ​mg/kg); (5) pep-PAPM@PTX (pep 6 ​mg/kg, PTX 10 ​mg/kg). Treatments were carried out every other day for 5 doses by i. v. injection via the tail vein. Antitumor activity was evaluated in terms of tumor volume, which was estimated as follows: tumor volume ​= ​a ​× ​b^2^/2, where a and b are the major and minor axes of tumors, respectively, as measured by a caliper. On day 14, the mice were sacrificed by cervical decapitation. The tumors and major organs were taken out from the sacrificed mice and weighted. For histopathological analysis, the excised tumors and major organs were fixed in 4% PBS-buffered paraformaldehyde, embedded in paraffin, and sectioned into 4-μm-thick slices. The sections were imaged under an optical microscope. For the analysis of CD8^+^ T cells infiltration in tumors, harvested tumors were ground in PBS and filtered through 75 ​μm filters. Collagenase Ⅰ and Ⅳ were added to the suspension to digest tumor tissues. After being washed with PBS three times, staining antibodies, including CD3^+^, CD4^+^, and CD8^+^, were incubated with cell suspension according to the manufacturer's instructions (Biolegend, USA). Stained cells were analyzed by flow cytometry. For cytokines determination, whole blood was extracted from the mice at the end of the treatment and centrifugated to obtain serum. IL-2 and IFN-γ were determined via ELISA kit.

### 2.11 Statistical analysis

Statistical analysis was performed with the SPSS statistics software. Data were reported as mean ​± ​SD. Statistically significant differences (∗*P* ​< ​0.05, ∗∗*P* ​< ​0.01) were determined by one-way ANOVA followed by Tukey's post-test.

## 3 Results and discussion

### 3.1 Synthesis and characterization of pep-PAPM@PTX

**Fig. 1:**
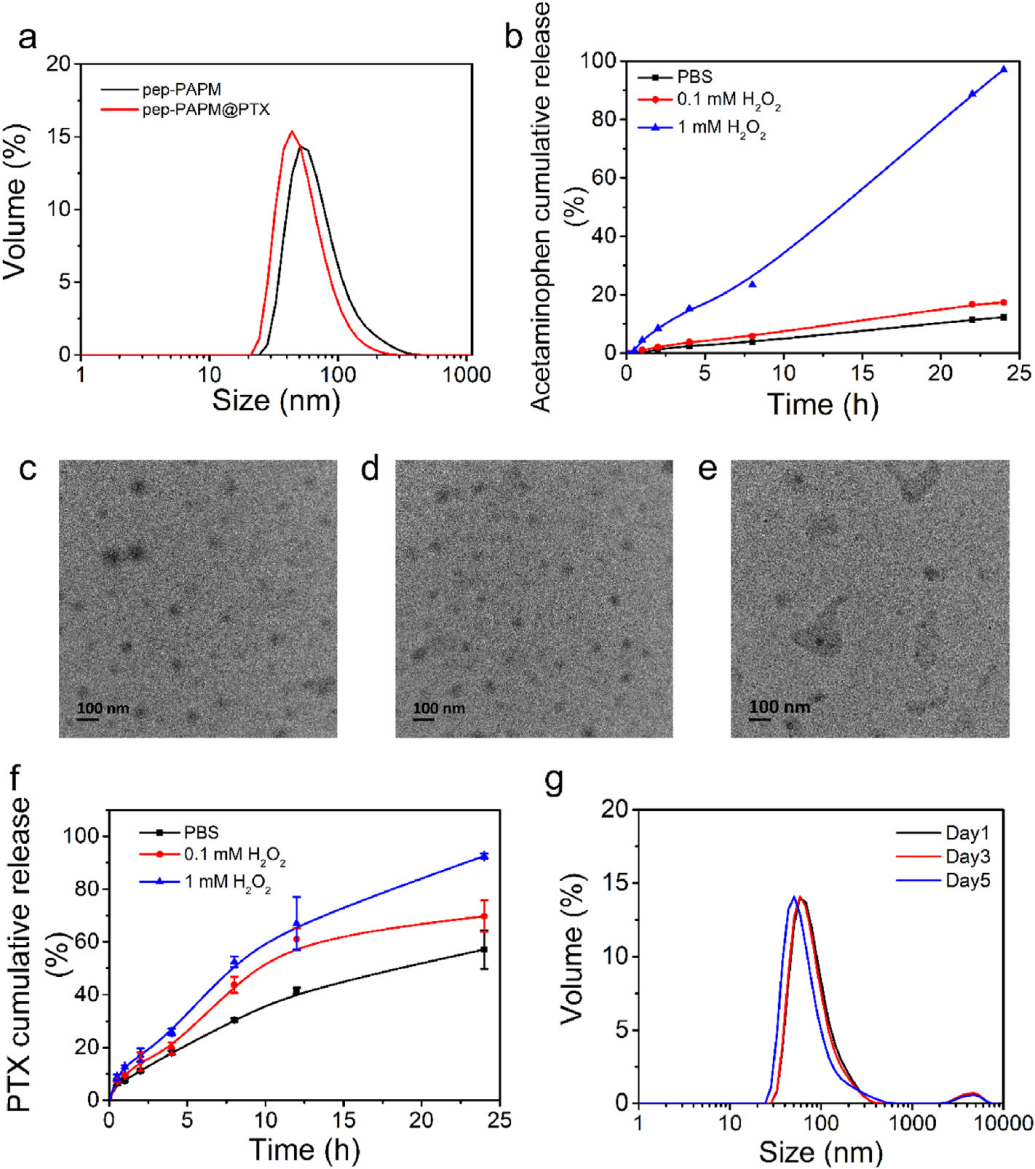
Characterization of pep-PAPM and pep-PAPM@PTX. (a) Size distributions of pep-PAPM and pep-PAPM@PTX micelles. (b) Cumulative release of acetaminophen from pep-PAPM at different H_2_O_2_ concentrations. (c–e) TEM images of pep-PAPM after 24 ​h incubation in the presence of (c) 0 ​mM ​(d) 0.1 ​mM (e) 1 ​mM ​H_2_O_2_. (f) PTX release profile from pep-PAPM@PTX in the presence of H_2_O_2_. (g) Colloidal stability of pep-PAPM@PTX in culture medium containing 10% FBS.

The synthetic route for the ROS-responsive amphiphilic block copolymer is shown in Scheme S1. Acetaminophen was conjugated with 2-hydroxyethyl methacrylate through a ROS-responsive thioether linker to give the AP monomer. Reversible addition-fragmentation chain transfer (RAFT) polymerization was employed to construct an amphiphilic block copolymer. First, allyl-terminated PEG reacted with 2-(Boc-amino)ethanethiol through a Michael addition reaction, followed by esterification with PETTC via DCC/DMAP method to produce a macromolecular CTA (BocNH-PEG-PETTC). BocNH-PEG-PETTC was then used to polymerize AP monomer, yielding a diblock copolymer PAP. This block copolymer was deprotected and terminally functionalized with a maleimide group to introduce a PD-L1 blockade peptide (CNYSKPTDRQYHF, pep) [21]through the thiol-maleimide “click” reaction (denoted as pep-PAP). According to ^1^H NMR (Figure S1-S4) and gel permeation chromatography (GPC) (Figure S5), both PAP and pep-PAP were successfully prepared. The critical micellization concentration (CMC) of pep-PAPM was 41 ​μg/mL (Figure S6). The pep-modified ROS-responsive micelle (pep-PAPM) was obtained by co-assembly of PAP and pep-PAP at a mass ratio of 4:1. PTX, as a model chemotherapy drug, was encapsulated into pep-PAPM by a nanoprecipitation method (pep-PAPM@PTX). The pep-PAPM and pep-PAPM@PTX micelles had diameters of 62 ​nm and 51 ​nm, as determined by dynamic light scattering (DLS) (Fig. 1a). The drug loading content and efficiency of PTX were calculated to be 7.2% and 71.8%, respectively.

The ROS-responsive drug release manners of pep-PAPM and pep-PAPM@PTX were investigated in the presence of H_2_O_2_. As shown in Fig. 1b, acetaminophen could be released slowly from pep-PAPM after incubation in PBS, with ∼10% released in 24 ​h. Treatment of 0.1 ​mM ​H_2_O_2_ slightly accelerated the drug release, while 24 h-incubation in the presence of 1 ​mM ​H_2_O_2_ led to an almost complete acetaminophen release. The β-thioether ester linkage could hardly be hydrolyzed due to the hydrophobic environment in the micelle core. However, upon oxidation of the thioether group to hydrophilic sulfone by H_2_O_2_, the neighboring ester bond would be more easily hydrolyzed [[25], [26], [27]], resulting in the fast release of free acetaminophen. Meanwhile, we observed the morphological transformation of pep-PAPM in response to H_2_O_2_ by TEM (Fig. 1c–e). The pep-PAPM micelles maintained their morphologies after 24 ​h incubation in the absence of H_2_O_2_, but they shrank slightly upon the addition of 0.1 ​mM ​H_2_O_2_. Moreover, exposure to 1 ​mM ​H_2_O_2_ drastically damaged the micelle structure. Accordingly, compared with the normal condition, pep-PAPM@PTX released PTX much faster in the presence of 1 ​mM ​H_2_O_2_, with over 90% of PTX released within 24 ​h (Fig. 1f). The fast release of PTX resulted from ROS-triggered dissociation of the micelle structure. We also evaluated the stability of pep-PAPM@PTX in a culture medium containing 10% fetal bovine serum (FBS). Notably, no significant change in particle size distribution was observed after 3-day of incubation, indicating micelles' high stability in the presence of serum (Fig. 1g).

### 3.2 Cellular uptake and cytotoxicity

**Fig. 2:**
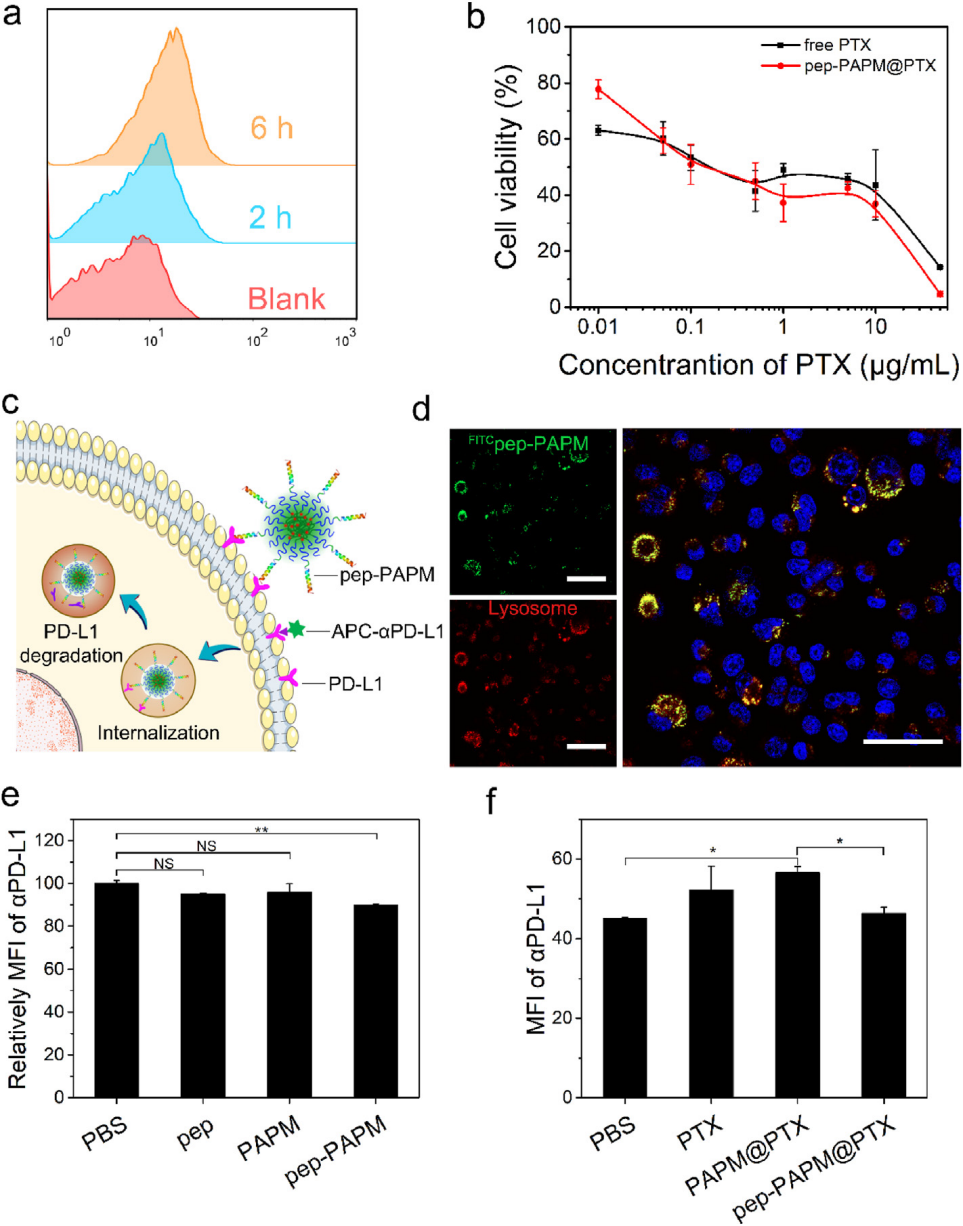
Cellular uptake and cytotoxicity of pep-PAPM@PTX and its possible mechanism of downregulating PD-L1 expression. (a) Time-dependent cellular uptake of pep-^FITC^PAPM by 4T1 cells measured by flow cytometry. (b) Cytotoxicity assays of PTX and pep-PAPM@PTX against 4T1 cells after 48 ​h treatment. (c) Schematic illustration of the multivalent binding of pep-PAPM towards PD-L1 to drive PD-L1 into lysosome degradation. (d) Colocalization of pep-^FITC^PAPM and lysosome after 4 ​h incubation with 4T1 cells. Nuclei stained with Hoechst 33,342 are shown in blue, lysosomes stained with LysoTracker Red are shown in red, and pep-^FITC^PAPM is shown in green. Scale bar: 50 ​μm. (e) The flow cytometric analysis of the cell surface PD-L1 in 4T1 cells treated by different groups for 1 ​h at 4 ​°C (pep eq. dose of 100 ​μg/mL). (f) The flow cytometric analysis of the cell surface PD-L1 in 4T1 cells treated by different groups for 24 ​h at 37 ​°C (PTX eq. dose of 20 ​μg/mL).

The cellular uptake of pep-PAPM by 4T1 cancer cells was observed using flow cytometry. To fluorescently label pep-PAPM, a fluorescein monomer (FITC-MA) was copolymerized with the AP monomer (Scheme S2). As shown in Fig. 2a, the fluorescence intensity of pep-^FITC^PAPM in 4T1 cells gradually increased with the incubation time, indicating a time-dependent endocytosis behavior. Subsequently, the in vitro cytotoxicity of pep-PAPM@PTX against 4T1 cells was investigated by MTT assay. Pep-PAPM alone had slight toxicity at a concentration of 100 ​μg/mL (Figure S7). The pep-PAPM@PTX micelles showed dose- and time-dependent cytotoxicity. The cytotoxicity of pep-PAPM@PTX was lower than free PTX at 24 ​h (Figure S8), probably due to the gradual release of PTX from micelles. However, prolonging the treatment time to 48 ​h significantly increased the cytotoxicity of pep-PAPM@PTX (IC50 value: 0.29 ​μg/mL), comparable to that of free PTX (IC50 value: 0.32 ​μg/mL) (Fig. 2b), because more PTX was released owing to the ROS-responsive property.

### 3.3 PD-L1 binding and downregulation

Given that the PD-L1 on the cancer cell surface can be restored quickly upon binding with antibodies or small molecular antagonists, it is crucial to interrupt the recycling or facilitate the degradation of PD-L1 intracellularly [13,28,29]. We hypothesized that the multivalent binding of the anti-PD-L1 peptide on the micelle surface with PD-L1 would drive its recycling pathway to lysosome degradation (Fig. 2c). The subcellular distribution of pep-^FITC^PAPM was visualized using confocal laser scanning microscopy (CLSM). As illustrated in Fig. 2d, the bright green fluorescence of pep-^FITC^PAPM was visible in the cells after 4 ​h incubation and highly colocalized with the red fluorescence of lysosomes, indicating that pep-PAPM could be transported into lysosomes.

To examine if pep-PAPM can bind onto cancer cells, we incubated 4T1 cells with pep-^FITC^PAPM at 4 ​°C, where cell-surface binding could occur, but cellular uptake was inhibited [30]. After incubation, a fluorescence-labeled PD-L1 antibody (APC-αPD-L1) was used to monitor the PD-L1 level on the cell surface, as determined by flow cytometry (Fig. 2e and S8). Compared with PBS, the surface PD-L1 level declined in cells treated by pep-PAPM, while no significant changes were detected in both pep and PAPM, suggesting that pep-PAPM was superior to free pep in binding PD-L1, which could be ascribed to the multivalent effect [31].

Subsequently, 4T1 cells were incubated with each formulation for 24 ​h at 37 ​°C to allow internalization, and the PD-L1 expression was detected by flow cytometry. As displayed in Fig. 2f and S9, free PTX can upregulate the expression of PD-L1 on tumor cells [11]. Notably, PAPM@PTX treatment induced a higher PD-L1 expression than free PTX, while pep-PAPM@PTX suppressed the PD-L1 expression significantly. These data indicate that pep-PAPM@PTX can reverse PTX-induced PD-L1 upregulation. The multivalent binding of pep-PAPM@PTX with PD-L1 directed PD-L1 to lysosomal degradation instead of recycling back to the cell surface, in line with the previous reports [20,32].

### 3.4 In vivo biodistribution

**Fig. 3:**
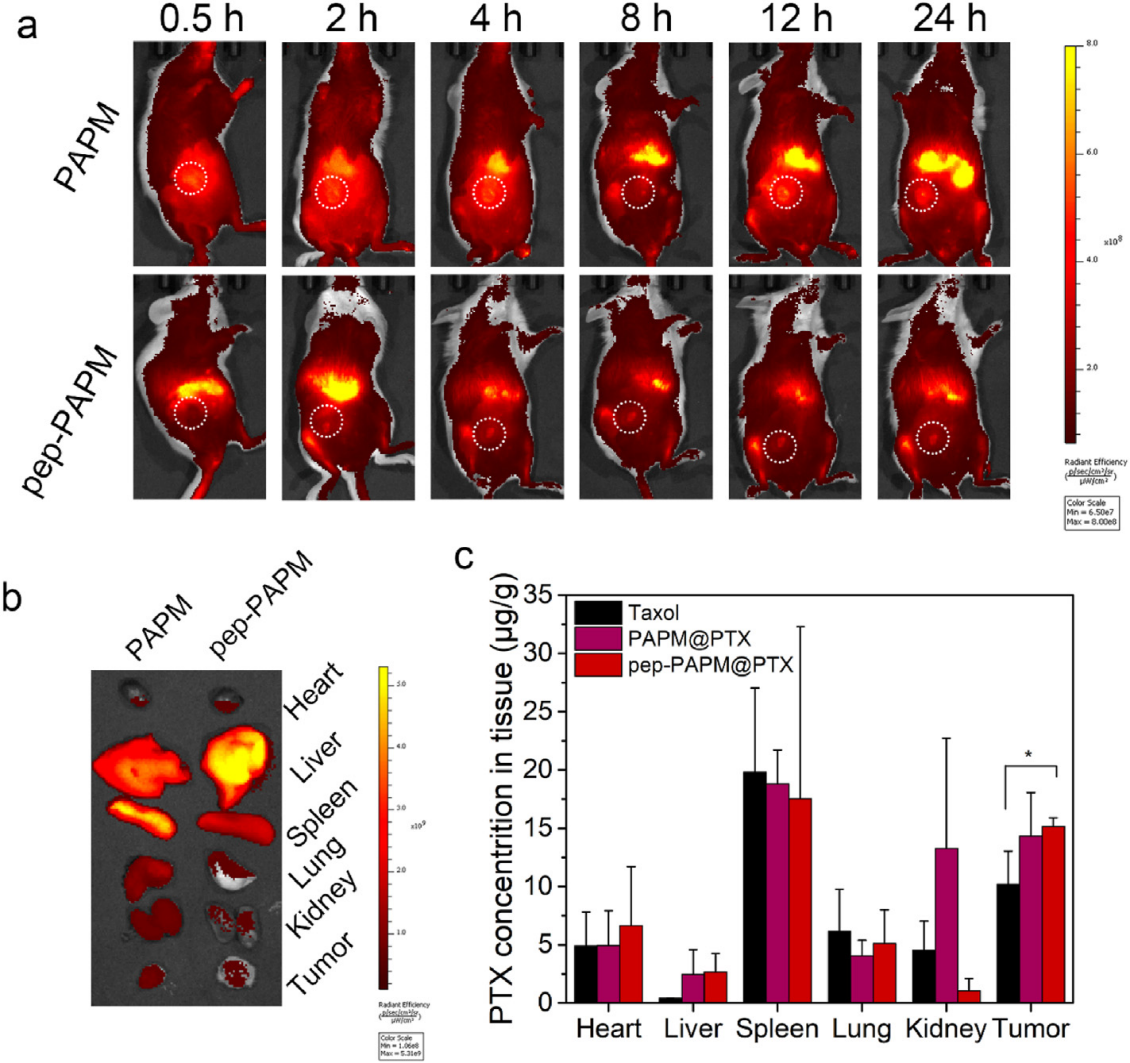
Biodistribution of pep-PAMP and pep-PAPM@PTX in vivo. (a) In vivo real-time imaging of 4T1 tumor-bearing mice after i. v. injection of Bodipy-labeled PAPM or pep-PAPM. The white circles indicate the tumor regions. (b) The ex vivo images of major organs and tumors of mice at 24 ​h post-treatment. (c) The PTX distribution in 4T1 tumor-bearing mice at 24 ​h after i. v. injection with Taxol, PAPM@PTX, or pep-PAPM@PTX (n ​= ​3).

The in vivo real-time imaging was performed on 4T1 tumor-bearing BALB/c mice after a single intravenous injection of Bodipy-labeled PAPM or pep-PAPM micelles (Fig. 3a). Intense fluorescence spread throughout the whole body and gradually accumulated in the tumor sites in both the ^Bodipy^PAPM and pep-^Bodipy^PAPM groups. Notably, the fluorescence signals of ^Bodipy^PAPM and pep-^Bodipy^PAPM declined slowly, suggesting a long blood circulation of micelles. Unexpectedly, pep modification of the micelles did not enhance the tumor accumulation of pep-^Bodipy^PAPM micelles as validated by the ex vivo imaging of dissected tumors (Fig. 3b). We confirmed this phenomenon in a CT26 tumor-bearing mouse model (Figure S11). Next, we investigated the in vivo distribution of PTX 24 ​h after treatment with Taxol, PAPM@PTX, or pep-PAPM@PTX. As shown in Fig. 3c, pep-PAPM@PTX treated mice had a 1.5-fold higher PTX accumulation in tumors than taxol-treated mice. The results indicate that the micellar drug delivery system improved tumor accumulation of PTX.

### 3.5 In vivo immune response and antitumor effect

**Fig. 4:**
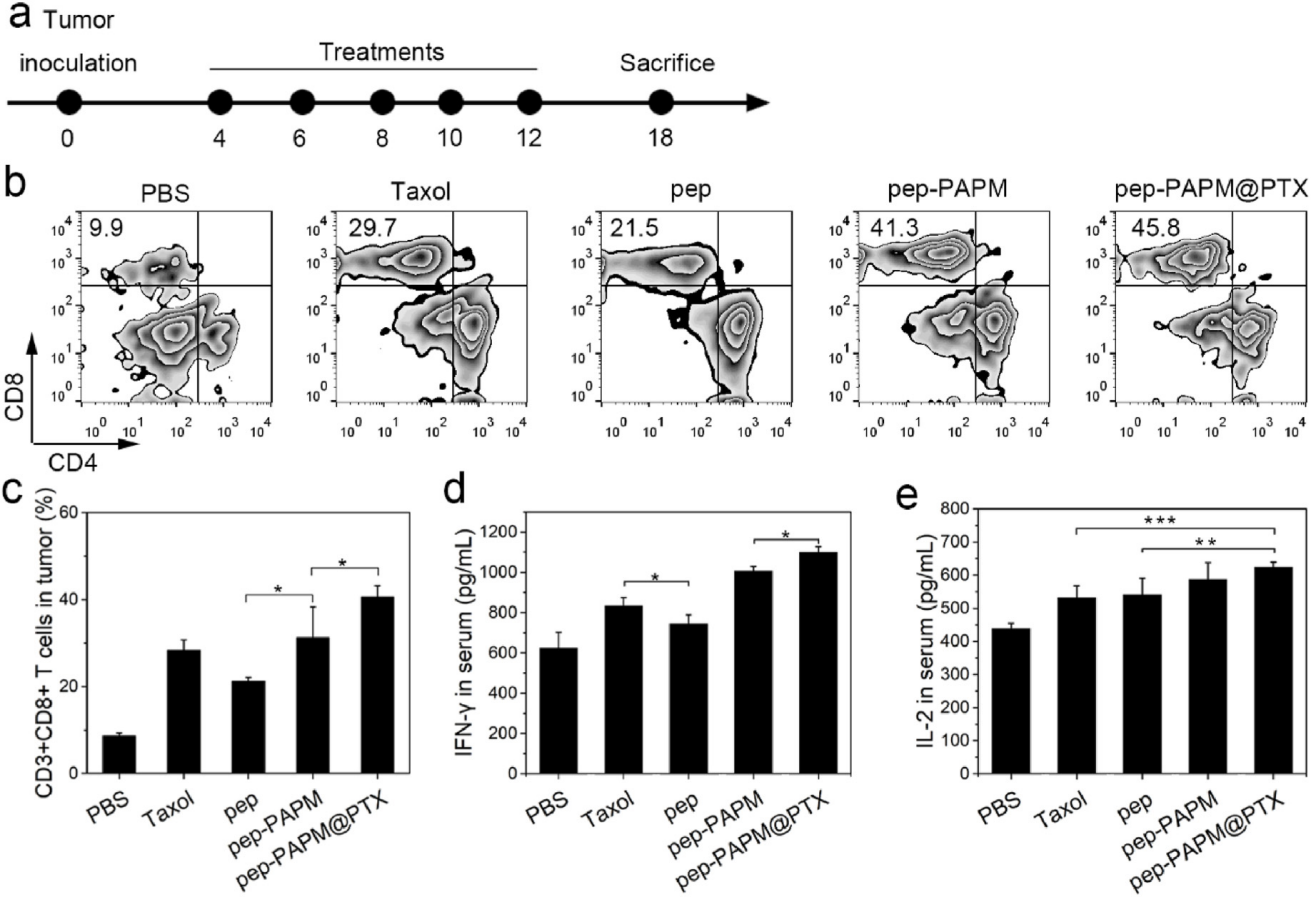
The improved immuno-microenvironment and systemic immunity induced by pep-PAMP@PTX. (a) Schematic of the development of tumor model and experiment design. (b,c) Flow cytometry analysis of in vivo lymphocytic infiltration in resected tumors; (b) the contour diagrams and (c) quantification. (d,e) The serum (d) IFN-γ and (e) IL-2 levels of mice determined on day 18.

We examined whether pep-PAPM@PTX promoted the antitumor immune response in vivo. The BALB/c mice bearing 4T1 tumors were administered with each formulation for 5 treatments. The tumor tissues were harvested 6 days after the last treatment to analyze the lymphocytic infiltrates, and the blood was collected to measure the cytokine levels (Fig. 4a). PTX significantly enhanced intratumoral infiltration of cytotoxic T lymphocytes (CTLs, CD3^+^CD8^+^ T cells) compared with the PBS group, probably due to its immunogenic cell death (ICD) inducing ability [33,34]. However, the upregulated PD-L1 expression would exhaust these T cells [35], thus weakening the antitumor immune response. Pep-PAPM micelles remarkably promoted intratumoral infiltration of CTLs, with a 1.47-fold higher than pep treatment. More importantly, pep-PAPM@PTX increased the CTLs infiltration to the utmost extent, resulting from the synergistic effect of PTX and multivalent pep (Fig. 4b and c). Meanwhile, the contents of inflammatory cytokines, such as IFN-γ and IL-2, in pep-PAPM@PTX-treated mice were markedly increased (Fig. 4d and e). These results demonstrate that pep-PAPM@PTX could activate the immune microenvironment and boost antitumor immune responses.

**Fig. 5:**
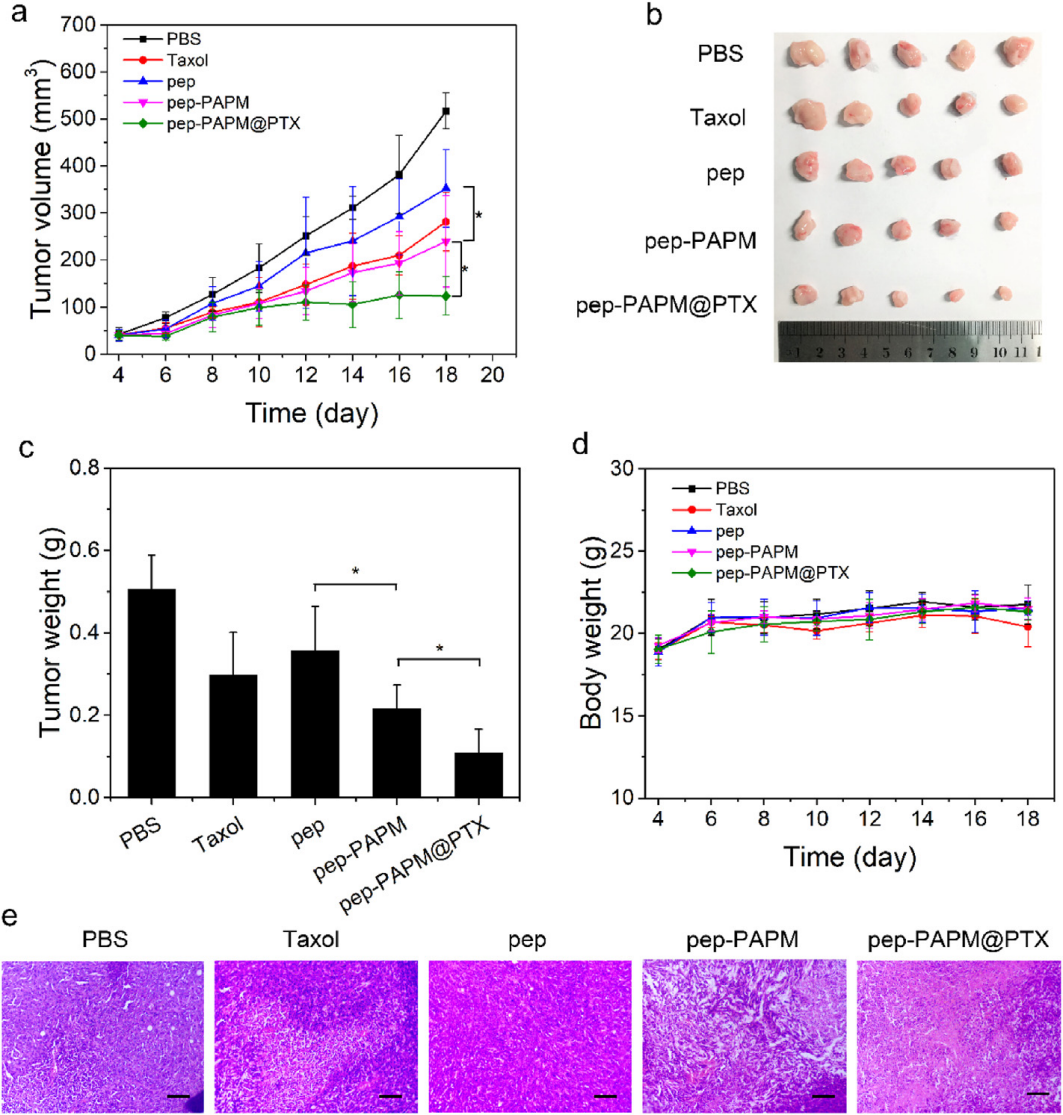
In vivo antitumor efficacy on a 4T1 breast cancer mice model. Once the tumor volumes reached ∼50 ​mm^3^, mice were intravenously injected with PBS, Taxol, pep, pep-PAPM, or pep-PAPM@PTX every other day for a total of 5 treatments (pep eq. dose of 6 ​mg/kg, PTX eq. dose of 10 ​mg/kg). (a) Tumor volume change of the mice after intravenous injection over time. (b) Image of resected tumors. (c) Weights of extracted tumors at the end of the treatment procedure. (d) Changes in body weight during the treatment. (e) H&E staining of tumors in different groups. Scale bar: 150 ​μm.

The in vivo antitumor activity of pep-PAPM@PTX was evaluated using a 4T1 breast tumor model. When the tumor volume reached about 50 ​mm^3^, BALB/c mice bearing 4T1 breast cancer were randomly divided into five groups. PBS, Taxol, pep, pep-PAPM, or pep-PAPM@PTX was administrated intravenously every other day for a total of 5 injections (PTX eq. dose of 10 ​mg/kg and pep eq. dose of 6 ​mg/kg). As shown in Fig. 5a and b, pep treatment slightly suppressed tumor growth compared to the PBS group. Taxol and pep-PAPM showed moderate tumor regression efficacy, while pep-PAPM@PTX remarkably inhibited tumor growth, with a significantly higher tumor inhibition rate (78%) than that of pep (30%), pep-PAPM (57%), and Taxol (41%) (Fig. 5c). Moreover, no significant changes in mice's body weight were observed (Fig. 4d). The H&E staining of tumor tissues supported the superior antitumor effect of pep-PAPM@PTX (Fig. 5e). More apoptotic and necrotic cells with nucleus shrinkage and fragmentation were found in pep-PAPM@PTX-treated tumors than those treated with PAPM, pep, or Taxol, demonstrating the improved antitumor efficacy of pep-PAPM@PTX. After treatment, no obvious histopathologic impairments were observed in the main organs, including the heart, liver, spleen, lung, and kidney (Figure S12). These results demonstrated the improved antitumor efficacy and biosafety of pep-PAPM@PTX.

## 4 Conclusions

In summary, we presented a ROS-responsive synergistic drug delivery system (pep-PAPM@PTX) for combining chemotherapy with immunotherapy. Pep-PAPM@PTX could crosslink the membrane PD-L1 and direct it from the recycling pathway to lysosomal degradation, downregulating PD-L1 expression and thus alleviating immunosuppression to CTLs. PTX can be readily released from micelles in response to elevated ROS levels inside tumor cells to exert pharmaceutical effects. The micelle-mediated combined chemo-immunotherapy exhibited improved therapeutic efficacy in a TNBC mouse model. We envision that this micellar drug delivery system provides a novel platform for an efficient combination of chemotherapy and ICB therapy.

## Author statement

**Doudou Hu**: Conceptualization, Methodology, Investigation, Writing – original draft, Writing – review & editing; **Wei Zhang**: Methodology, Investigation; **Jiajia Xiang**: Supervision, Writing – review & editing, Funding acquisition; **Dongdong Li**: Methodology; **Yong Chen**: Investigation; **Pengcheng Yuan**: Investigation; **Shiqun Shao**: Methodology, **Zhuxian Zhou**: Methodology; **Youqing Shen**: Supervision; **Jianbin Tang**: Supervision, Project administration, Funding acquisition.

## Declaration of competing interest

The authors declare that they have no known competing financial interests or personal relationships that could have appeared to influence the work reported in this paper.
