# Supplementary material for: A ROS-responsive synergistic delivery system for combined immunotherapy and chemotherapy
Source: Mater Today Bio. 2022 May 11;14:100284. doi: 10.1016/j.mtbio.2022.100284 (PMC9130108; doi:10.1016/j.mtbio.2022.100284)
Supplement: Multimedia component 1 [file mmc1.docx]

**Supporting Information**

**A ROS-responsive Synergistic Delivery System for Combined Immunotherapy and Chemotherapy**

Doudou Hu^1,3,4^†, Wei Zhang^1,2,3^†, Jiajia Xiang^1,2,3^*, Dongdong Li^1,2,3^, Yong Chen^1,2,3^, Pengcheng Yuan^1,2,3^, Shiqun Shao^1,2,3^, Zhuxian Zhou^1,2,3^, Youqing Shen^1,2,3^, and Jianbin Tang^1,2,3^*

^1^ Zhejiang Key Laboratory of Smart BioMaterials and Center for Bionanoengineering, College of Chemical and Biological Engineering, Zhejiang University, Hangzhou 310027, China

^2^ ZJU-Hangzhou Global Scientific and Technological Innovation Center, Hangzhou 311215, China

^3^ Key Laboratory of Biomass Chemical Engineering of the Ministry of Education, College of Chemical and Biological Engineering, Zhejiang University, Hangzhou 310027, China

^4^ Subtropical Sericulture and Mulberry Resources Protection and Safety Engineering Research Center, College of Animal Science, South China Agricultural University, Guangzhou, Guangdong, 510642, China

† Equal contribution.

* Corresponding authors.

Email addresses: xiang_jj@zju.edu.cn (J. Xiang), jianbin@zju.edu.cn (J. Tang)

**Supplementary figures**


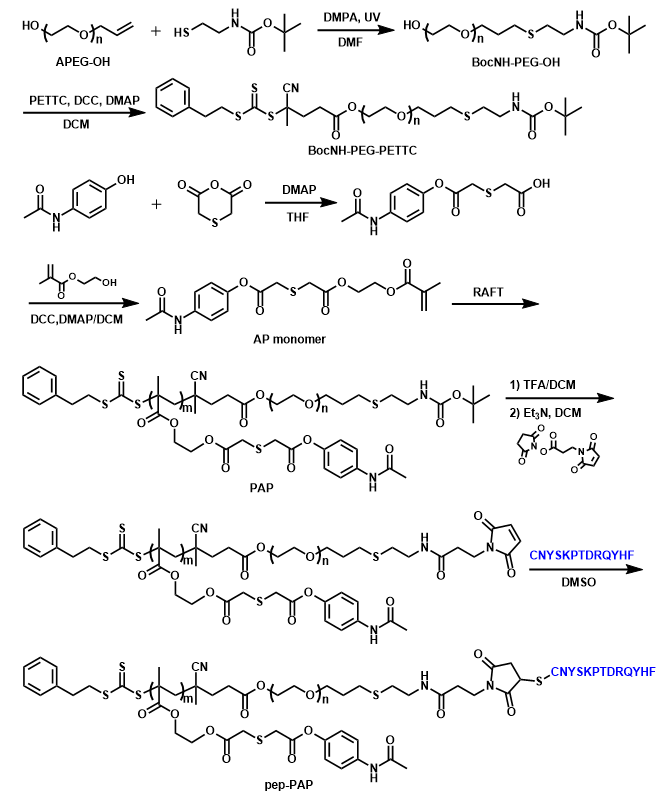


**Scheme S1.** The synthetic route of PAP and pep-PAP.


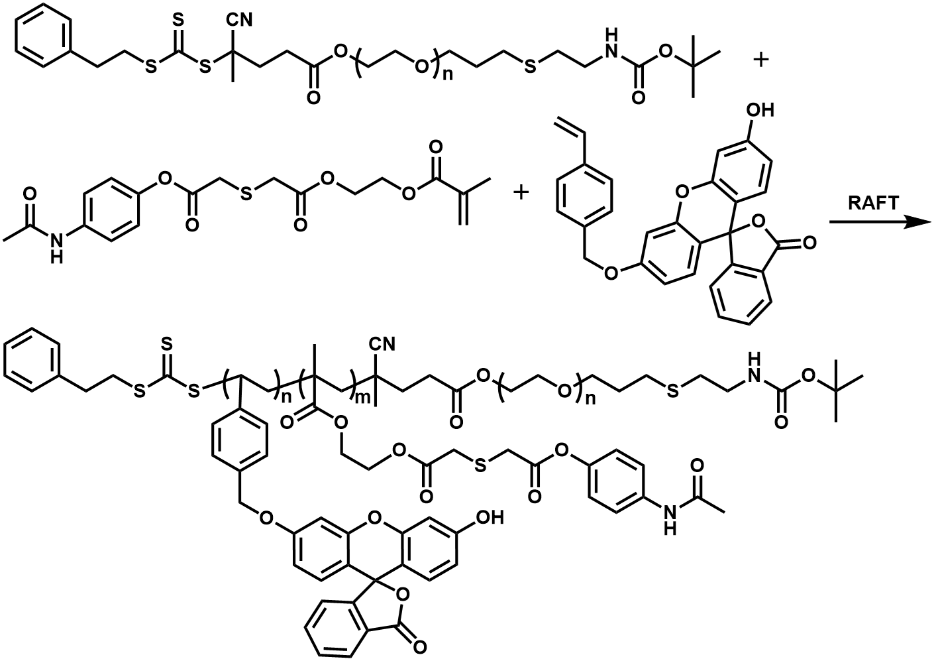


**Scheme S2.** The synthetic route of FITC-labeled PAP.


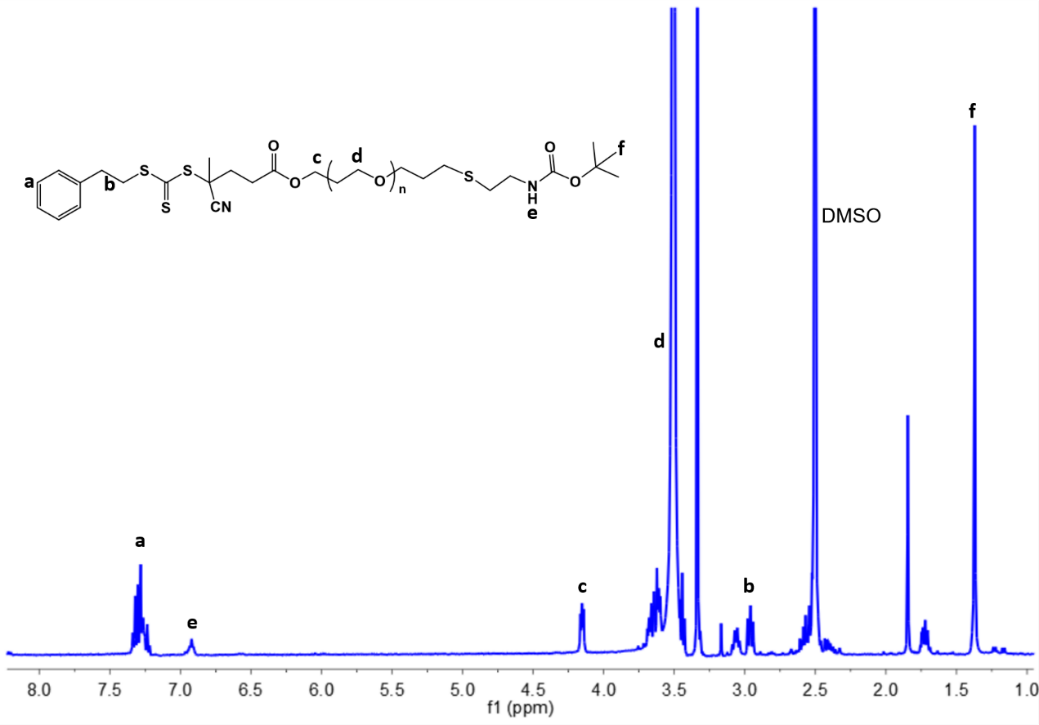


**Figure S1.** The ^1^H NMR spectrum of PEG_2K_-PETTC in DMSO-d6.


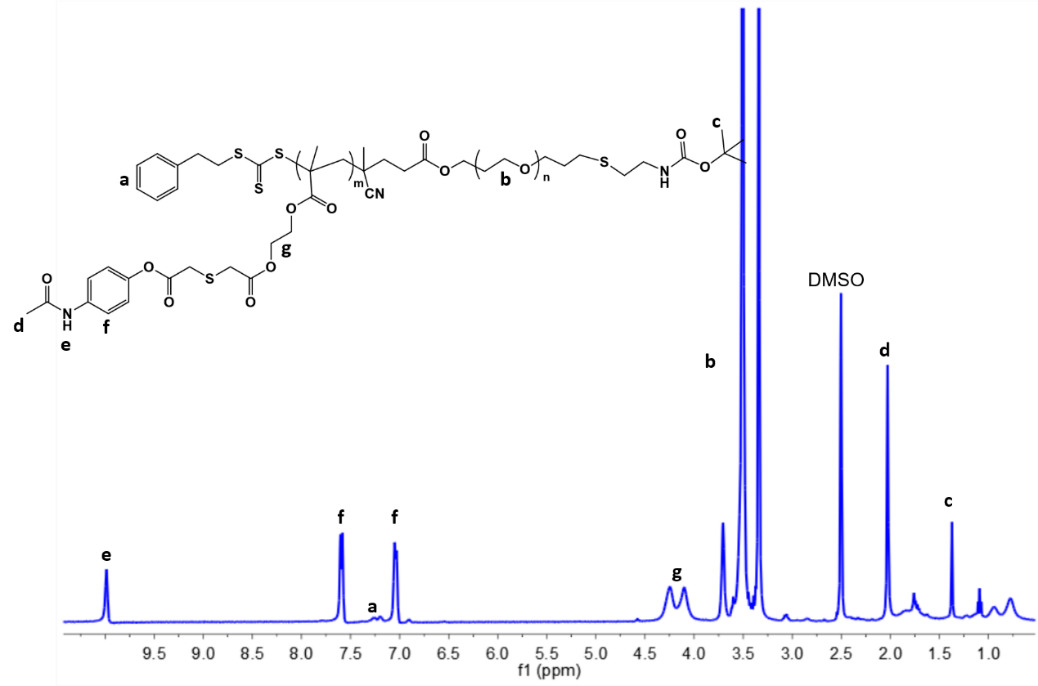


**Figure S2.** The ^1^H NMR spectrum of PAP in DMSO-d6.


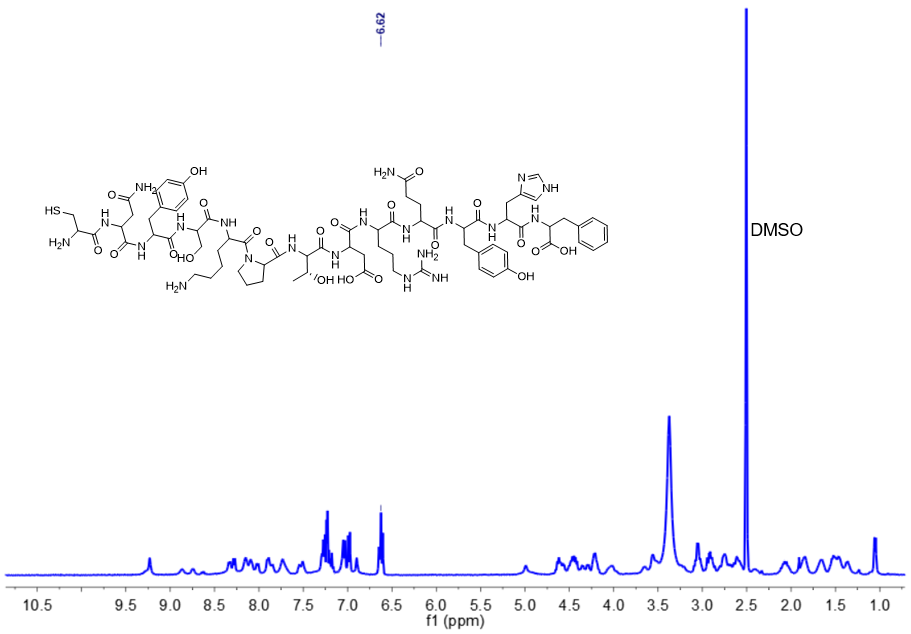


**Figure S3**. The ^1^H NMR spectrum of pep in DMSO-d6.


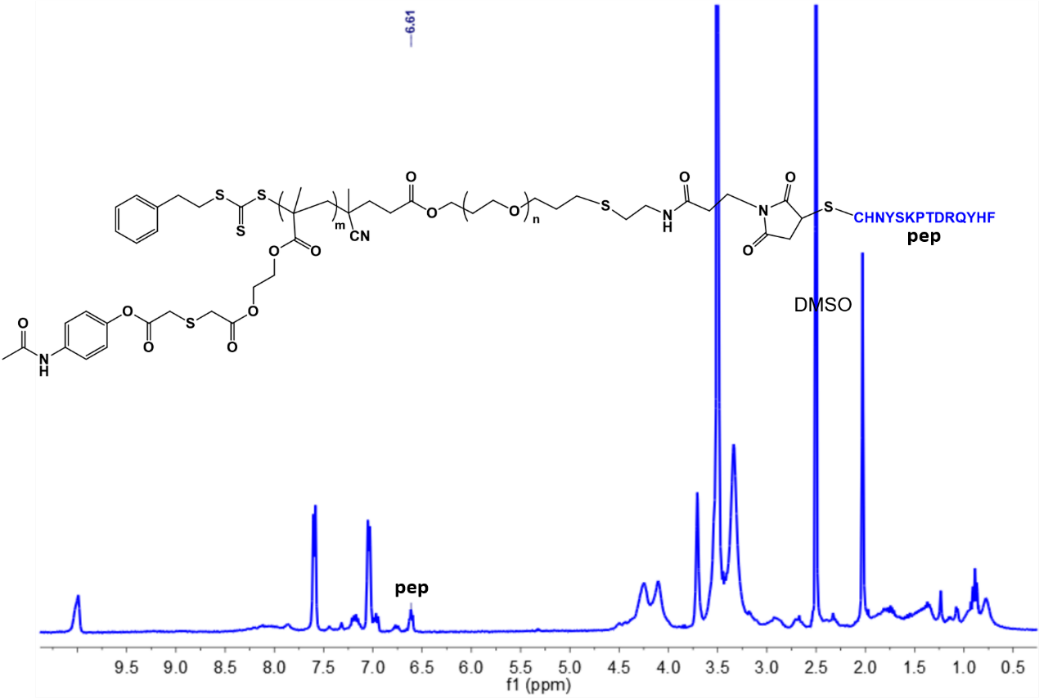


**Figure S4**. The ^1^H NMR spectrum of pep-PAP in DMSO-d6.





**Figure S5.** GPC traces of PAP and pep-PAP (DMF, 50 °C, 0.8 mL min^-1^).





**Figure S6.** The fluorescence intensities of Nile red as a function of the pep-PAPM concentrations.





**Figure S7.** Cytotoxicity of pep-PAPM on 4T1 cells at 24 h.





**Figure S8.** Cytotoxicity of pep-PAPM@PTX against 4T1 cells after 24 h incubation.


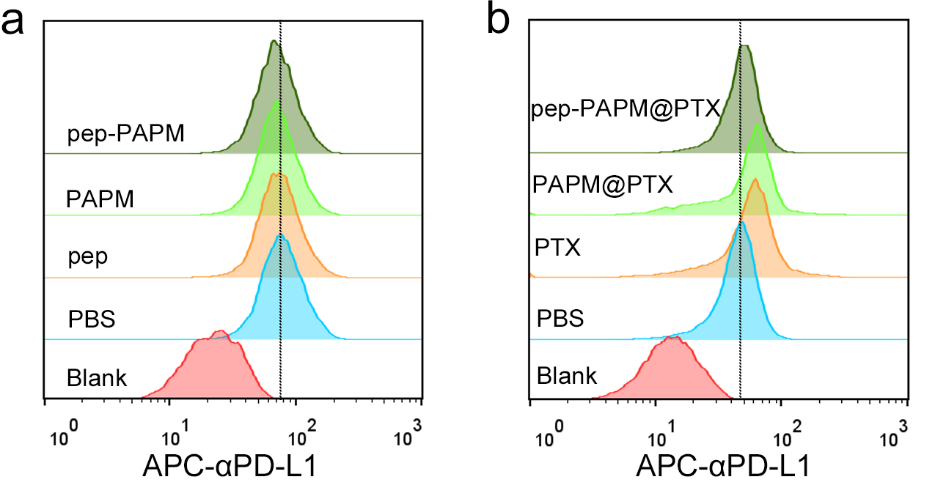


**Figure S9**. The flow cytometry chart of the cell surface PD-L1 in 4T1 cells treated with each formulation for 1 h at 4℃ (pep eq. dose of 100 μg/mL).


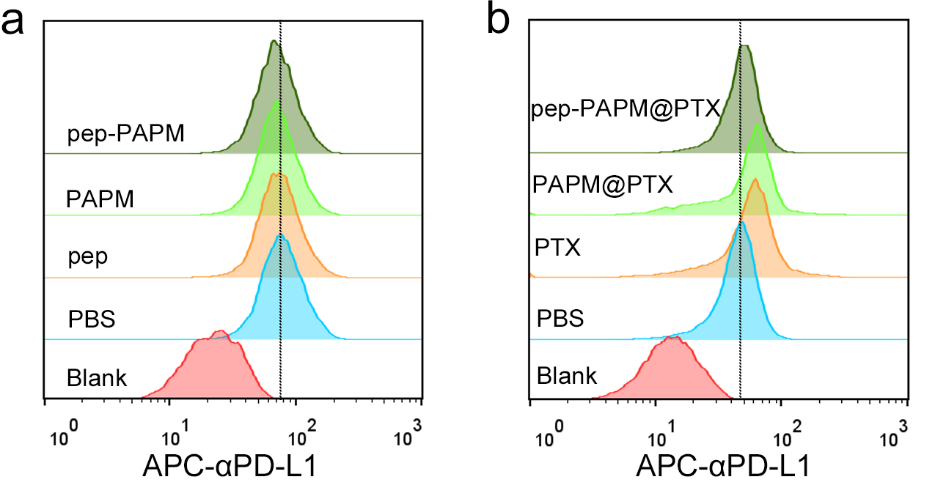


**Figure S10**. The flow cytometry chart of the cell surface PD-L1 in 4T1 cells treated with each formulation for 24 h at 37℃ (PTX eq. dose of 20 μg/mL).


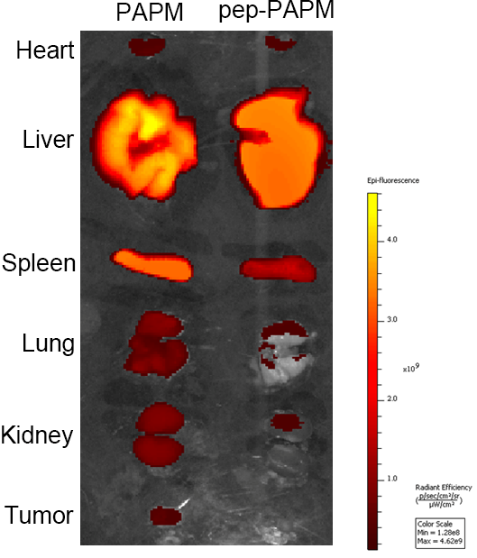


**Figure S11.** Ex vivo images of major organs and tumors from CT26 tumor-bearing mice 12 h post-treatment.


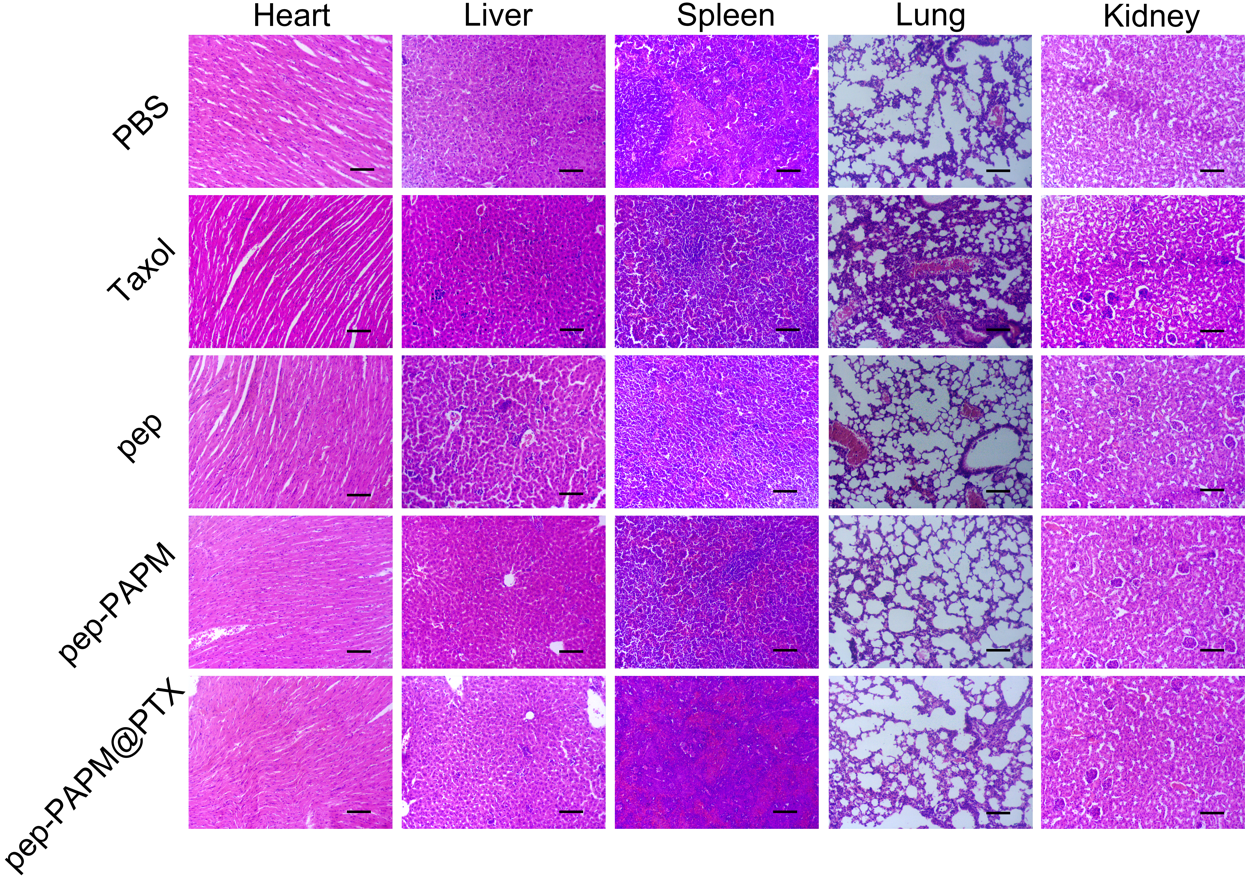


**Figure S12.** H&E-stained images of the heart, liver, spleen, lung, and kidney from different groups. Scale bar: 150 μm.
